# Antioxidant Activity and Other Characteristics of Lactic Acid Bacteria Isolated from Korean Traditional Sweet Potato Stalk Kimchi

**DOI:** 10.3390/foods13203261

**Published:** 2024-10-13

**Authors:** Jung-Min Park, Ji-Woon Moon, Bo-Zheng Zhang, Byoung-Ki An

**Affiliations:** 1 Department of Food Marketing and Safety, Konkuk University, Seoul 05029, Republic of Korea; pjm0321@konkuk.ac.kr (J.-M.P.); nym21@naver.com (J.-W.M.); slurpee24@163.com (B.-Z.Z.); 2 Animal Resources Research Center, Konkuk University, Seoul 05029, Republic of Korea

**Keywords:** kimchi, DPPH activity, lactic acid bacteria, microbial community, antimicrobial activity

## Abstract

The aim of this study was to examine the biological activity and probiotic properties of lactic acid bacteria (LAB) isolated from sweet potato stalk kimchi (SPK). Various LAB and *Bacillus* spp. are active in the early stages of the fermentation of kimchi made from sweet potato stalk. Four strains of LAB were identified, including SPK2 (*Levilactobacillus brevis* ATCC 14869), SPK3 (*Latilactobacillus sakei* NBRC 15893), SPK8 and SPK9 (*Leuconostoc mesenteroides* subsp. *dextranicum* NCFB 529). SPK2, SPK3, SPK8, and SPK9 showed 64.64–94.23% bile acid resistance and 78.66–82.61% pH resistance. We identified over 10^6^ CFU/mL after heat treatment at 75 °C. Four strains showed high antimicrobial activity to *Escherichia coli and Salmonella* Typhimurium with a clear zone of >11 mm. SPK2 had the highest antioxidative potentials, higher than the other three bacteria, with 44.96 μg of gallic acid equivalent/mg and 63.57% DPPH scavenging activity. These results demonstrate that the four strains isolated from sweet potato kimchi stalk show potential as probiotics with excellent antibacterial effects and may be useful in developing health-promoting products.

## 1. Introduction

In recent years, sweet potato stalk (Convolvulaceae; *Ipomoea batatas* L.) has become one of the key crops in the world. Sweet potatoes are produced in over 100 countries [1,2] owing to their agronomic and nutritional advantages. With 54.97% of the world’s annual production, China is the largest producer of sweet potatoes. [3,4]. In Republic of Korea, consumption has increased, with an annual production of 329,516 and 348,912 tons in 2013 and 2023, respectively [5].

Sweet potatoes are an abundant source of energy and contain many valuable by-products, such as leaves, stems, stalks and tuber [6]. Sweet potatoes are therefore a rich source of carbohydrates, fiber, beta-carotene, minerals and other nutrients [7]. Additionally, edible plants contain many phenolic compounds that have antioxidant activities and other health-beneficial properties. In recent years, studies have been carried out to determine the health benefits of the of sweet potato stalk. The tuberous root can be processed into starch, flour, and alcoholic drinks, and the leaves into kimchi, because the tips of sweet potato leaves are highly nutritious [8]. Used as a vegetable in Africa, China and Japan, sweet potato stalk and petioles are high in protein, increasing their potential as a source of protein for livestock. The stalks have physiological activities, such as antioxidant and antibacterial properties [9]. The polyphenol content of sweet potato stalk is higher than that of sweet potato roots, flesh, and peel [10]. Although many studies have been conducted on the physiological activity of sweet potato stalk and various products, there is a lack of research on adapting fermented foods such as sweet potato stem kimchi.

As kimchi is fermented by over 100 different LAB, it has been highlighted as a rich source of LAB. Kimchi is particularly dependent on the microflora that determines the antimicrobial and antioxidant activity of kimchi, lactic acid bacteria such as *Lactobacillus*, *Leuconostoc*, *Pediococcus* and *Weissella*. Many studies have reported the beneficial effects of LAB—anticancer, immunostimulatory effects—enhancing immunity and protecting against intestinal pathogens [11,12]. In addition, LAB not only improve the shelf life of kimchi, but also contribute desirable flavors and aromas as a food additive [13].

Therefore, this study was the first attempt to produce kimchi using sweet potato stem, which is not widely used or is discarded in Korea. In this study, we aimed to examine the possibility of using sweet potato stalk as a novel, healthy, functional food material and provided basic data on lactic acid bacteria identification as well as antioxidant, antimicrobial, and metabolic fresh fermentation stages to evaluate the characteristics of sweet potato stalk kimchi.

## 2. Materials and Methods

### 2.1. Preparing Sweet Potato Stalk Kimchi

The Jeollanam-do province in Republic of Korea purchased the sweet potato stems. Ten kilograms of peeled sweet potato stalk were marinated in 10% salt for 1 h. Subsequently, they were blanched in boiling water for 3 min, rinsed under running water, washed in cold water, and left to drain for 1 h.

Minor materials consisting of garlic (2.8%), ginger (1.0%), anchovy fish sauce (2.5%), and pepper powder (3.3%) were added to make kimchi. All materials were produced in Korea, purchased from a major market (Seoul, Republic of Korea) located in the Gwangjin Gu of Seoul, and transported to the lab within 1 h. The experiments were performed in triplicate and stored at 4 ℃ prior to analysis. In the study, the pH and acidity of fresh fermented kimchi were 5.69 and 0.435% the first time. To select the optimal ripening stage, samples were selected, respectively, after taking measurements every week during the kimchi fermentation process. We then selected 6-week fermented sweet potato stem kimchi with a pH of 4.19 and an acidity of 0.765 as the sample. In general, the optimal pH at the ripening stage for eating kimchi is 4.2–4.4, and the acidity is approximately 0.6% [14,15].

### 2.2. Sampling, pH, and Total Acidity

The sweet potato stalk samples were isolated for 6 weeks and fermented in a refrigerator for the storage of sweet potato stalk kimchi. Ten grams SPK were added to 90 mL sterile distilled water, mixed with stomacher (Stomacher^®^ 400 circulator; Seward Inc., Worthing, West Sussex, UK), and filtered using papers (Whatman plc, Kent, UK). The pH value was measured with a pH meter (pH Basic+; Sartorius AG, Göttingen, Germany). A homogeneous solution was filtered using filter paper (Whatman plc, Kent, UK), and NaOH (0.1 N, Duksan, Seoul, Republic of Korea), required to neutralize the required amount of kimchi solution (10 mL), was used to convert the total lactic acid content to set the acidity (%, *w*/*v*) by titration three times.

### 2.3. Sample Collection and Bacteria Isolation

A sweet potato stalk kimchi sample was used to isolate lactic acid bacteria (LAB). All samples were gathered in sterile containers and were screened for the presence of LAB. Twenty-five grams of the sample was suspended in 225 mL of sterile saline solution, and tenfold dilutions were prepared from the solution. The diluted solution was inoculated onto MRS agar (De Man, Rogosa and Sharpe Agar, Difco, BD, Detroit, MI, USA) and incubated for 24 h at 30 °C. LAB-like colonies were selected based on morphology and size and were purified by plating on MRS agar plates. The 12 isolates were stored at −80 °C in MRS as glycerol stocks. They were then propagated in MRS broth at 30 °C for 24–48 h.

### 2.4. Antimicrobial Properties of Bacteria Isolated from Sweet Potato Kimchi

An antimicrobial test was conducted with 12 colonies isolated from sweet potato kimchi and incubated at 30 °C for 48 h in MRS broth. As described by Wu et al. [16], antagonism against various bacterial pathogens was evaluated with slight modifications. The strains used for antibacterial activity were those related to food poisoning: *Escherichia coli* (*E. coli*) KCCM 21052, *Salmonella* Typhimurium p99 (*Sal.* Typhimurium), *Staphylococcus aureus* (*St. aureus*) KVCC BA1100335, and *Listeria monocytogenes* (*Lis. monocytogenes*) KVCC BA0001449.

The paper disc method was used to determine the antibacterial activity of the compounds produced by each isolate [17]. For each pathogen, a paper disc was inoculated with 50 µL of a bacterial suspension containing roughly 10^7^ CFU/mL isolated from sweet potato stalk kimchi. The paper discs were then incubated at 37 °C for 48 h and antibiotic activity was determined by measuring the diameter of the clear zones. Based on these results, we selected four excellent lactic acid bacteria.

### 2.5. Acid Tolerance of Selected Strains

Acid pH tolerance experiments were performed as described by Archer et al. [18] with minor modifications. MRS broth (99 mL of MRS broth inoculated with 1 mL of each of the four superior antimicrobial lactic acid bacterial isolates) was adjusted to pH 2.5 with 1 N HCl and 1 N NaOH, inoculated with a 5% incubated bacterial suspension and grown anaerobically at 37 °C for 24 h. Aliquots were taken after 4 h, diluted and plated on MRS agar plates. Conventional non-acidified MRS medium (pH 6.2) was used as a control. After 48 h of anaerobic incubation at 37 °C, the number of viable colonies was the count [19].

The survival rate was calculated as described [20]:

Resistance percentage = (viable cell counts in MRS broth at a pH of 2.5/viable cell counts in MRS broth at a pH of 6) × 100%

### 2.6. Bile Salt Tolerance of Selected Strains

A total of 0.85% NaCl was added to the pellet of LAB (*w*/*v*); then, MRS broth containing 0.3% bile salt was inoculated with each LAB. After incubation of the suspended broth at 37 °C for 3 h, viable cells were counted. Uninoculated MRS broth supplemented with the respective bile salt was used as a control.

Survival rate (%) = (cell number in MRS containing bile salts/cell number in MRS) × 100

### 2.7. Heat Resistance of Selected Strains

The MRS broth (99 mL of MRS broth inoculated with 1 mL of each of the four superior antimicrobial lactic acid bacterial isolates) was incubated for 18 h at 30 °C, and 10 mL aliquots were placed in sterile capped glass test tubes. They were incubated in a shaking water bath at 37, 55, 65 and 75 °C for 10 min, and the colonies were counted after incubation on MRS plates at 37 °C for 48 h. Viable cell counts in the fresh culture and cultures after different heat treatments were analyzed using the standard plate count method.

### 2.8. Molecular Identification of LAB and Phylogenetic Tree Construction of Selected Strains

Lactic acid bacteria strains have been extracted from kimchi made from the stem of sweet potatoes using MRS agar. Genomic DNA colonies from sweet potato stalk kimchi were isolated from plates exhibiting antibacterial activity using a PowerPrep DNA Extraction (Kogene Biotech, Seoul, Republic of Korea), with changes. Polymerase chain reaction amplification of the 16S rRNA gene was performed using the universal primer 27F (5’-AGAGTTTGATCCTGGCTCAG-3,’ forward) and 1492R (5’-GGCTACCTTGTTTACGACTT-3,’ reverse). The PCR product was verified by loading the sample on a 1.5% agarose gel containing 100 mg/mL etidium bromide (EtBr) and electrophoresing the amplicon. Further identification was conducted with 16S rRNA gene sequence analysis from SolGent (SolGent Co., Ltd., Daejeon, Korea). BLAST (https://blast.ncbi.nlm.nih.gov/Blast.cgi, accessed on 20 February 2024) was used to compare the gene sequences of the selected extracts with those available in the databases. Phylogenetic trees were constructed using the neighbor-joining method (https://www.megasoftware.net/ (accessed on 20 February 2024)).

### 2.9. Library Construction and Sequencing of Sweet Potato Kimchi

The DNA sample was extracted using a DNeasy PowerSoil Kit (Qiagen, Hilden, Germany) according to the manufacturer’s instructions. Quant-IT PicoGreen (Invitrogen) was used to quantify extracted DNA. The 16S rRNA genes of the distinct regions (16S V4) were amplifications with primers (V3-TCGTCGGCAGCGTCAGATGTGTATAAGAGACAGCCTACGGGNGGCWGCAG; V4-TCGTCGGCAGCGTCAGATGTGTATAAGAGACAGCCTACGGGNGGCWGCAG). The procedure is the following: initial denaturation at 95 °C for 3 min and 25 cycles of 30 s at 95 °C, 30 s at 55 °C and 30 s at 72 °C, followed by a final extension at 72 °C for 5 min. The initial PCR product was purified using beads. The second PCR was purified beads. Finally, the purified product was qualified by qPCR using the qPCR Qualification guide (KAPA Library Quantification kits for Illumina sequencing platforms) and a ScreenTape and TapeStation D1000 (Agilent Technologies, Waldbronn, Germany).

### 2.10. Data Processing and Amplicon Sequence Variant (ASV) Analysis

After sequencing, samples were grouped by index sequence, and paired-end FASTQ files were generated for each sample. After sequencing, samples were grouped by index sequence, and paired-end FASTQ files were generated for each sample along with a pre-processing step, which removed the forward (Read 1) and reverse (Read 2) sequences. To correct errors that occurred during amplicon sequencing, the DADA2 (v1.18.0) package in R (v4.0.3) was used. Chimeric sequences were removed, and ASVs were made using the consensus method of DADA2, after which the paired-end sequences (which corrected the sequencing error in the previous step) were assembled into one sequence. Amplicon sequence variants of less than 350 base pairs were removed using R v4.0.3. The ASV sequence was subjected to BLAST+ (v2.9.0) against the reference DB (NCBI 16S Microbial DB), and taxonomic content was assigned based on the organisms with the highest 85% similarity.

### 2.11. Antioxidant Activity of Sweet Potato Stalk Kimchi

#### 2.11.1. Preparation of Sample

The method described by the authors Lin and Chang [21] was used in a modified form. We incubated four strains on MRS broth at 37 °C for 16–18 h, then centrifuged the sample at 3000× *g* for 10 min at 4 °C. The supernatant was removed, the same volume of phosphate buffered saline (PBS) was added and centrifuged as described above, and the supernatant was used as a sample.

#### 2.11.2. Measuring 2,2-Diphenyl-1-Picrylhydrazyl Radical Scavenging Activity

The radical scavenging activity of DPPH was evaluated as described before [22,23], with some changes. We mixed 2,2-diphenyl-1-picryhydrazyl (Sigma) and ethanol (Samchun, Korea), then prepared a 0.3 mM DPPH solution and used it for the experiment. Briefly, the sample (2.5 mL) was reacted with 1 mL of the DPPH reagent and incubated in the dark at 25 °C for 30 min. The absorbance of the sample was then measured. The absorbance of the control was 0.3 mM DPPH solution. Absorbance was measured at 517 nm (UV-1601, Shizuoka, Japan).

DPPH radical scavenging activity was calculated as follows:

DPPH radical scavenging activity = 100 − [(Abs_sample_ − Abs_blank_) * 100]/Abs_control_

Abs, Absorbance

Sample: sample 2.5 mL + 0.3 mM DPPH reagent 1 mL

Blank: supernatant 2.5 mL + ethanol 1 mL

Control: 0.3 mM DPPH reagent 1 mL + ethanol 2.5 mL

#### 2.11.3. Determination of Total Polyphenols

The Folin Ciocalteu colorimetric method [22,23] was used to determine the total polyphenol content of sweet potato kimchi [24,25]. Briefly, an aliquot (100 μL) was mixed with 700 μL of water. Folin–Ciocalteu’s phenol reagent (Sigma-Aldrich, Inc., St. Louis, MO, USA) (500 μL) was then added. After 2 min, 150 μL Na_2_CO_3_ (20% *w*/*v*) was added to the mixture and allowed to react at 37 °C for 2 h at room temperature. A UV spectrophotometer was used to measure the absorbance at 765 nm (Beckman Coulter, Du 530, Anaheim, CA, USA). A gallic acid equivalent (GAE) standard calibration curve was constructed.

The equation for the GAE, with an R2 value of 0.992, was as follows:

Total polyphenols content = 0.031 * OD + 0.159

### 2.12. Statistical Analysis

Measurements were conducted in triplicate and expressed as mean ± standard error. Statistical analyses were performed using the SPSS program (SPSS version 12.0, SPSS, Chicago, IL, USA) with unpaired t-tests and repeated measures ANOVA where appropriate. When significant, differences in means were determined with Duncan’s multiple range test (*p* < 0.05). Correlations between the mean values were determined using Pearson’s correlation tests.

## 3. Result and Discussion

### 3.1. Antimicrobial Activity from Sweet Potato Stalk Kimchi

Twelve colonies of different sizes and characteristics were isolated from sweet potato stalk kimchi on MRS agar and used as samples. Twelve LAB strains that were extracted from kimchi made from sweet stem potatoes were screened for antimicrobial activity against foodborne pathogens, including *E. coli*, *St. aureus*, *Sal.* Typhimurium, and *Lis. monocytogenes*. Among them, we have selected four strains with an antibacterial effect and have numbered them as 2, 3, 8 and 9. The results in Table 1 show that the LAB’ strains among the bacterial isolates from sweet potato stalk kimchi samples showed the highest antibacterial activity against foodborne pathogens such as *E. coli* and *Sal.* Typhimurium. The average inhibition zone diameter ranged from 11 to 15 mm (Table 1).

It is suggested that these LAB strains may inhibit the growth of foodborne pathogens in the sweet potato stalk kimchi during kimchi fermentation. This means that LAB would manufacture inhibitory substances, such as organic acids, carbon dioxide, hydrogen peroxide and bacteriocins, during the fermentation process [26]. In addition, they can decrease the potential for redox, water activity, and nutrient depletion [27]. Thus, the inhibitors produced by LAB inhibit pathogenic and spoilage microorganisms [28]. However, in our study, *St. aureus* and *Lis. monocytogenes* showed the lowest growth inhibition among all pathogens tested. *E. coli* and *Salmonella* Typhimurium were more sensitive than *St. aureus* and *Lis. monocytogenes*. This is consistent with Ammora et al.’s [29] study, where Gram-negative bacteria are more sensitive than Gram-positive bacteria. Meanwhile, other studies have shown that the cell-free supernatant of LAB was bioactive against various pathogens, including *St. aureus*, *E. coli*, *Lis. monocytogenes*, and *Sal*. typhimurium [30,31,32,33].

In this study, LAB from isolated sweet potato stalk kimchi showed, during the fermentation process, a strong in vitro inhibition of the gram-negative foodborne pathogens tested. Therefore, importantly for food safety and a functional effect, LAB isolated from sweet potato stalk kimchi can be used as a natural additive.

### 3.2. pH Tolerance

The original pure stomach pH of a human being ranges from 1.4 to 2.0; therefore, most microorganisms are expected to be killed [34], although it is thought that the pH of the stomach can be slightly raised by ingested food. The LAB has to be able to survive in the stomach so that the lactic acid bacteria can perform various physiological functions in the body and gastrointestinal tract and colonize the intestine [35]. The acid resistance of this strain is considered an important characteristic because it allows it to survive and remain functional after gastrointestinal transit. Therefore, acid resistance is an important factor for the quality stabilization of fermented foods and for use as a functional health food factor [36].

The results of this study show that the survival of the four LAB strains isolated from sweet potato stalk kimchi was between 78.66% to 82.61% when exposed to pH 2.5 for 3 h. The highest survival rate (82.61%) was observed in SPK 3 (*Lacilactobacillus sakei* strain NBRC 15893), followed by SPK 2 (*Levilactobacillus brevis* ATCC 14869), SPK 8 and SPK 9 (*Leuconostoc mesenteroides* subsp. *dextranicum* NCFB 529) (Table 2). The survival rate of LAB varies depending on the strain; however, a decrease of 1–2 log usually occurs. This indicated that the activity of the isolated strain was relatively stable.

Therefore, although the viable bacterial count of the four bacteria decreased after 3 h of shaking in artificial gastric fluid compared to the initial viable bacterial count, the survival rate was a relatively high resistance compared to other strains. For example, *Lactobacillus lactis* isolated from soybean paste shows a survival rate of 36.1% at a pH of 2.5 and a survival rate of 72.0% in a bile acid environment of 0.3% [37]. It is suggested that the LAB isolated from sweet potato stalk kimchi can be stored in the body and can survive and reach the small intestine. The results of this study are consistent with previous findings that LAB from fermented foods are able to maintain viability when exposed to pH ranging from 1.0 to 3.0 [38,39].

### 3.3. Bile Salt Reference

Bile acid tolerance is a characteristic required for survival in the small intestine. Bile acid is a substance secreted by the duodenum that destroys the cell membrane of bacteria and inhibits their growth; therefore, it must be resistant to bile to function as a probiotic [40]. It is also thought that it can only survive in the human stomach and intestines if it is present in bile acids at concentrations above 0.3%. SPK 2 had a log 5.74 compared with the total bacterial count after the addition of 0.3% bile acid for 3 h of incubation. SPK 3, SPK 8, and SPK 9 had a log 6.25, 6.16, and 6.13, respectively with the addition of 0.3% bile acid. Although this result showed that the total bacterial count was slightly suppressed in the 0.3% bile acid condition, this study showed a high survival rate of SPK 2 at 84% and SPK 9 at 94%, indicating excellent resistance to bile salts.

Cholesterol-degrading strains are resistant to bile salts and have a good ability to deconjugate them [41]. Therefore, SPK 2 and SPK 9 selected in this study did not appear to be inhibited, even in media containing 0.3% oxgall, and are thought to have potential for cholesterol degradation (Table 2).

### 3.4. Heat Resistance

**Figure 1:**
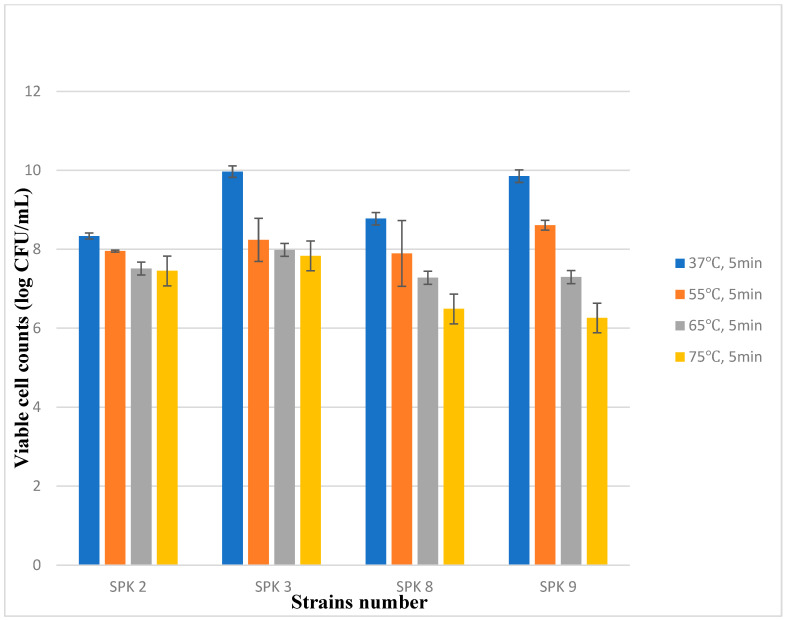
Survivals of strains that are treated for 5 min at 37, 55, 65 and 75 °C, after separating the strains from the sweet potato stalk kimchi. All values are expressed as the mean ± standard deviation of the three replicates. SPK 2, *Levilactobacillus brevis* ATCC 14869; SPK 3, with *Latilactobacillus sakei* strain NBRC 15893; SPK 8 and SPK 9, *Leuconostoc mesenteroides* subsp. *dextranicum* strain NCFB 529.

Four bacterial strains, isolated from sweet potato stalk kimchi, were incubated at 37, 55, 65, and 75 °C for 10 min, and the change in the microbial count was evaluated (Figure 1). SPK 2 showed a relative survival of 89.54–95.55% under 55, 65, and 75 °C; SPK 3 showed 79.96–82.57% under 55, 65, and 75 °C; while SPK 8 and 9 showed 74.00–89.97% and 63.55–87.41% survival, respectively, compared with those at 37 °C. For strains SPK 3, SPK 8, and SPK 9, viable cell counts at 75 °C were lower than those at 37 °C, demonstrating the enhanced thermotolerance of SPK 2 when cultured at 75 °C compared with other strains. All four isolated strains showed lower survival rates with an increasing temperature compared to 37 °C (control), which is closest to the growth temperature. Because the optimal growth temperature for most LAB is between 30 and 45 °C, previous studies have described reduced survival at higher temperatures as well [42,43,44,45]. Similarly, activity decreases with treatment time, but four LAB isolated from sweet potato stalk kimchi show a relatively high heat stability [46]. Another study reported that some thermophilic strains are viable at temperatures of up to 60 °C, and *Lactobacillus acidophilus* and *Streptococcus thermophilus* can grow maximally at temperatures of up to 45 °C [47,48,49]. This may also depend on the type of lactic acid bacteria in the kimchi, fermentation mechanism, and fermentation time.

### 3.5. Identification of Isolated Bacteria

**Figure 2:**
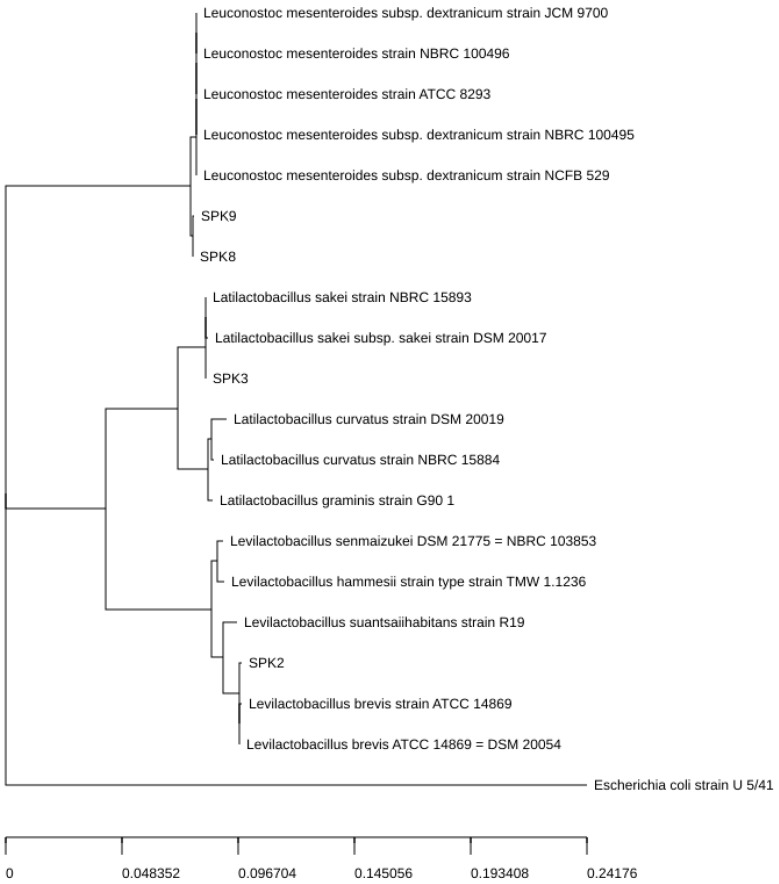
Phylogenetic tree construction using neighbor-joining method and gene sequences, based on 16S rRNA sequencing, showing the positions of strain samples and other closely related lactic acid bacteria isolated from sweet potato stalk kimchi.

Four sample lactic acid bacteria were chosen for 16S rDNA sequencing based on their antimicrobial activity. The 16S rDNA sequencing revealed that the SPK 2 strain showed a homology to sequences of 99.86% homogeneity with *Levilactobacillus brevis* ATCC 14869, SPK 3 strain showed the highest homology to sequences of 100% with *Latilactobacillus sakei* strain NBRC 15893, and SPK 8 and SPK 9 strains showed a homology to sequences of 99.79%–99.86% with *Leuconostoc mesenteroides* subsp. *dextranicum* strain NCFB 529 (Figure 2).

### 3.6. Microbial Community Analysis

**Figure 3:**
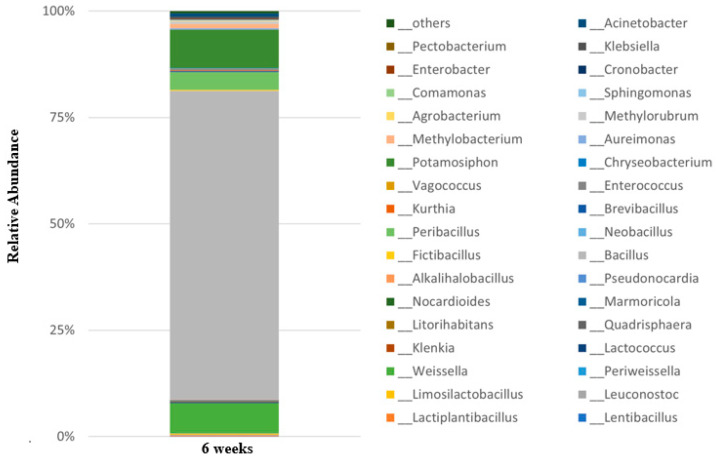
Taxonomic profile of sweet potato stalk kimchi at the genus level. The bar plot indicates the microbial community of all microorganisms observed in sweet potato kimchi.

According to the Shannon indices, sweet potato stalk kimchi displayed the highest diversity (3.041), indicating that its microbial community of sweet potato stalk kimchi was stable. A total of 192 an amplicon sequence variant (ASVs) were identified, and the main microbial composition of sweet potato stalk kimchi had relative abundance of over 0.05 (Figure 3). A total of 192 microbial operational taxonomic unit (OTU) sequences were clustered into 10 phyla and 36 genera. *Bacillus* spp. were dominant (71.70%), followed by *Potamosiphon* sp. (8.9%) and *Weissella* sp. (6.9%). Among the strains, first, *Bacillus* spp. were the most abundant (71.7%), and *Bacillus subtilis* was the most dominant species (67.7%) in sweet potato stalk kimchi. *B. subtilis* has more benefits as a probiotic than other species [50] and non-spore-forming bacteria such as *Lactobacillus*, *Bifidobacterium*, *Leuconostoc*, and *Pediococcus* species [51]. 

Second, *Potamosiphon australiensis* contains a phycoerythrin-rich protein, which is one of the exciting colours that can be obtained from genera of red algae and cyanobacteria [52]. The red-violet protein has antimicrobial and antioxidant properties [53], making it a new and interesting bioresource for the production of novel high-end products for nutraceutical applications. Lastly, *Weissella* sp. is a gram-positive bacterium that encompasses a phylogenetically coherent group of LAB [54], with Weissella confusa, found in fermented foods such as sausages and rice wine, and suggested to be a probiotic [55,56]. Among the LAB identified during kimchi fermentation, *Weissella*, *Pediococcus, Lactobacillus*, and *Leuconostoc* species play key roles in kimchi fermentation [57,58]. In particular, the potent probiotic strain of *Weissella confusa* confers several other human health benefits [59]. Besides that in this study, the microbial community that plays a role in kimchi fermentation was identified; *Leuconostoc* (0.27%), *Lactococcus* (0.25%), *Limosilactobacillus* (0.25%), *Lactiplantibacillus* (0.15%), *Lentibacillus* (0.08%), and *Periweissella* (0.07%) in sweet potato stalk kimchi [60]. Genomically characterizing these LBs may facilitate the potential use of strains of this genus as starter cultures in sweet potato stem kimchi.

### 3.7. DPPH Radical Scavenging

Free radicals play a key role in biological harm, and DPPH is known to measure the free radical scavenging activity of antioxidants [61]. Therefore, antioxidant activity was measured to determine the effect of fermented foods on selected bacteria from sweet potato stalk kimchi.

**Figure 4:**
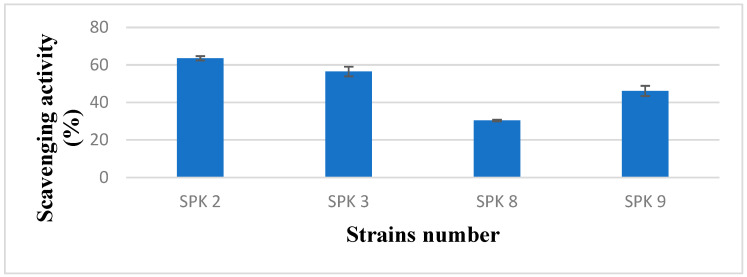
DPPH radical scavenging activity for strains. All values were mean ± standard deviations of triplicates. SPK 2, *Levilactobacillus brevis* ATCC 14869; SPK 3, with *Latilactobacillus sakei* strain NBRC 15893; SPK 8 and SPK 9, *Leuconostoc mesenteroides* subsp. *dextranicum* strain NCFB 529.

The DPPH free radical scavenging activity of the *Levilactobacillus brevis* (SPK 2) was the highest at 63.47%, while those of *Latilactobacillus sakei* (SPK 3) and *Leuconostoc mesenteroides* subsp. *dextranicum* (SPK 8 and 9) varied from 30.37% to 56.45% (Figure 4). This is due to the high content of polyphenolic compounds, anthocyanins, and the potency or concentration of reducing substances, primarily phenolic compounds, vitamin C, and phenolic acid, which are capable of scavenging DPPH free radicals [62,63,64].

Anthocyanins, which have complex patterns of anthocyanidin glycosylation, and acylation, are one of the most important polyphenols The content of anthocyanin is strongly affected by the composition of purple-fleshed sweet potato, which contains an acylated construction, and among sweet potato anthocyanin, anthocyanin with a caffeoyl group had a strong effect on antioxidant activity. In addition, another study reported that diacylated anthocyanins had higher antioxidant activity in sweet potato anthocyanins [65,66].

This is also because the antioxidant activity increases by neutralizing the oxygen radicals generated by the phenolic compounds, such as ferulic acid, present in sweet potato. The antioxidant capacity of kimchi varies depending on the type of LAB isolated [14]. Therefore, sweet potato stalk kimchi demonstrates antioxidant properties by scavenging DPPH, and could be a potential candidate for reducing oxidative stress-related diseases in humans. Sweet potato stalk also showed relatively higher DPPH radical scavenging activity than vegetables described by other researchers [67]. Sweet potato stem kimchi has antioxidant and antibacterial properties, which may be helpful in the development of various food matrices and pharmaceutical products.

### 3.8. Total Phenolic Content

Polyphenols have a strong reducing activity owing to their hydrogen-donating ability, electron-donating ability, radical stabilization, and metal-chelating activity. Additionally, the hydroxyl group has a hydrogen-donating ability, showing a strong antioxidant ability [68]. 

The total phenolic content results for sweet potato stalk kimchi are presented in Table 3. In the case of *Levilactobacillus brevis* (SPK 2), *Latilactobacillus sakei* (SPK 3) and *Leuconostoc mesenteroides* subsp. *dextranicum* (SPK 8 and 9), the concentrations were 44.96, 22.53, 34.76, and 29.56 µg GAE/mg, respectively. The relationship between total phenolic content and DPPH activities was also studied by Pearson correlation analysis (−0.02493 to 0.74952). The total phenolic content showed a positive correlation with the DPPH assay, except for *Latilactobacillus sakei* (SPK 3). In another study, the total phenolic content of fermented sweet potato stalk kimchi was higher than that of raw materials (i.e., raw sweet potato and red mustard leaves) [69].

This is thought to be because ethyl and vinyl derivatives of phenol, which have antioxidant activity, are produced by microorganisms and heating reactions as fermentation progresses [70,71]. In general, various enzymes produced during fermentation decompose cellulose or lignin to increase the extraction of phenolic compounds [72]. 

Also, it is believed that several enzymes produced during the fermentation process convert glycosides into non-glycosides and decompose proteins to produce aromatic amino acids. Aromatic amino acids are treated differently from phenolic contents, but they are also reported to be beneficial to health as substances that reduce oxidative stress in the body through radical scavenging ability and chelating effects [73].

Kim reported that the total polyphenol content of sweet potato tip shoots was 8.48 to 42.69 mg GAE/g [74]. Also, the buds contained the stems of sweet potato, with a content of 9.05 to 32.69 mg GAE/100 g fw.

Phenolics are not evenly distributed throughout the plant, and their distribution and levels depend on a number of factors, including the plant part and the environment [75,76]. Sweet potato stalk contains a much higher total polyphenol content than any other commercial vegetable, including sweet potato storage roots and potato tubers [77,78]. Thus, phenolics are important functional components of sweet potato stalk, and their antioxidant activity is beneficial to human health [79,80]. In view of global trends, more diverse research is needed because all parts of the sweet potato are presented as an important food that can face distant future conditions involving food supply problems, especially in developing countries.

## 4. Conclusions

Recently, sweet potato stalk kimchi and its potential as a probiotic have gained significant attention owing to its health benefits. In this study, we evaluated the microbial community distribution, the antimicrobial activities, and the antioxidant effects of lactic acid bacteria in sweet potato stalk kimchi. At 6 weeks of fermentation, the bacterial community of sweet potato stalk kimchi was dominated by *Bacillus* spp. (71.70%), followed by *Potamosiphon* sp. (8.9%) and *Weissella* sp. (6.9%). Moreover, we identified excellent LAB isolated from sweet potato stalk kimchi, such as *Levilactobacillus brevis* ATCC 14869 (SPK 2), *Latilactobacillus sakei* NBRC 15893 (SPK 3), and *Leuconostoc mesenteroides* subsp. *dextranicum* NCFB 529 (SPK 8 and 9). We suggest that these strains display excellent antioxidant and antimicrobial activities.

Therefore, we believe that physiologically active substances secreted by LAB in sweet potato stalk kimchi could be developed into diverse natural antimicrobial agents and antioxidant functional foods for widespread use in the food industry. The results of this study provided useful information on the basic characteristics and functionality of sweet potato stem kimchi, adapted to the food fermentation industry.

## Figures and Tables

**Table 1:** Antimicrobial activity against pathogens of four strains.

**Strains**	Negative Control	Species Clear Zone Surrounding the Disc (mm)			
		*Escherichia coli*	*Staphylococcus aureus*	*Salmonella Typhimurium*	*Listeria monocytogenes*
2	-	+++	-	+	-
3	-	++	-	++	-
8	-	++	-	+	-
9	-	+	-	++	-

*Escherichia coli* KCCM 21052; *Salmonella* Typhimurium P99; *Staphylococcus aureus* KVCC BA1100335; *Listeria monocytogenes* KVCC BA0001449; Negative control: distilled water; +++: Strong inhibition zone (≥15 mm); ++: clear inhibition zone (≥12 mm, <15 mm); +: slight inhibition zone (<10 mm); -: No inhibition.

**Table 2:** Low pH and bile salt tolerance of lactic acid bacterial strains.

Strains	Tolerance to Low pH (log CFU/mL)			Tolerance to Bile Salt (log CFU/mL)		
	Initial Population	After 3 h	Survival Rate (%)	0% Bile Salt	0.3% Bile Salt	Survival Rate (%)
2	10.39 ± 0.31 ^a^	8.40 ± 0.10 ^a,b^	81.28 ^a,b^	6.82 ± 0.04 ^c^	5.74 ± 0.03 ^a^	84.15 ± 0.52 ^b^
3	10.31 ± 0.07 ^a^	8.52 ± 0.05 ^a^	82.61 ^a^	9.67 ± 0.47 ^a^	6.25 ± 0.60 ^a^	64.64 ± 5.83 ^d^
8	10.47 ± 0.08 ^a^	8.38 ± 0.08 ^a,b^	79.99 ^b,c^	8.72 ± 0.10 ^b^	6.16 ± 0.24 ^a^	70.59 ± 2.02 ^c^
9	10.51 ± 0.12 ^a^	8.26 ± 0.05 ^b^	78.66 ^c^	6.50 ± 0.06 ^c^	6.13 ± 0.08 ^a^	94.23 ± 0.93 ^a^

Values are mean ± standard deviation (*n* = 3). Means in the same column and with different lower-case letters (a–d) indicate significant difference (*p* < 0.05).

**Table 3:** Total phenolic contents of sweet potato stalk kimchi; µg of gallic acid equivalent/mg.

Strains	Total Phenol Content (µg of GAEs/mg Extract)
SPK 2	44.96 ± 0.29
SPK 3	22.53 ± 0.28
SPK 8	34.76 ± 1.29
SPK 9	29.56 ± 0.36

All values were mean ± standard deviation of triplicates. SPK 2, *Levilactobacillus brevis* ATCC 14869; SPK 3, with *Latilactobacillus sakei* strain NBRC 15893; SPK 8 and SPK 9, *Leuconostoc mesenteroides* subsp. *dextranicum* strain NCFB 529.

## Data Availability

The original contributions presented in the study are included in the article, further inquiries can be directed to the corresponding author.
